# Supplementary material for: Modeling spinal locomotor circuits for movements in developing zebrafish
Source: eLife. 2021 Sep 2;10:e67453. doi: 10.7554/eLife.67453 (PMC8492062; doi:10.7554/eLife.67453)
Supplement: Transparent reporting form [file elife-67453-transrepform.docx]

***eLife’s* transparent reporting form**

We encourage authors to provide detailed information *within their submission* to facilitate the interpretation and replication of experiments. Authors can upload supporting documentation to indicate the use of appropriate reporting guidelines for health-related research (see [EQUATOR Network](http://www.equator-network.org/%20)), life science research (see the [BioSharing Information Resource](https://biosharing.org/)), or the [ARRIVE guidelines](http://www.plosbiology.org/article/info:doi/10.1371/journal.pbio.1000412) for reporting work involving animal research. Where applicable, authors should refer to any relevant reporting standards documents in this form.

If you have any questions, please consult our Journal Policies and/or contact us: [editorial@elifesciences.org](mailto:editorial@elifesciences.org).

**Sample-size estimation**

- You should state whether an appropriate sample size was computed when the study was being designed
- You should state the statistical method of sample size computation and any required assumptions
- If no explicit power analysis was used, you should describe how you decided what sample (replicate) size (number) to use

Please outline where this information can be found within the submission (e.g., sections or figure legends), or explain why this information doesn’t apply to your submission:

Sample size estimation was done for sensitivity testing of the beat-and-glide model using a significance level of 0.05 and a desired power of 0.8. The means, 95% confidence interval and standard deviations of episode duration, inter-episode interval, delay at peak autocorrelation of tail beat and minimum of left-right cross-correlation of the base model (control) were calculated and used in the power analysis. Estimated means for significant differences were outside of the 95% confidence interval of the means of the above parameters of the base model

For sample size estimation for knockout simulations, duration of wash-in and wash-out epochs was based on a significance level of 0.05 and a desired power of 0.8. The means and standard deviations of episode duration and inter-episode intervals of the base model (control) during the pre-wash-in epoch were calculated. Estimated means for significant differences were outside of the 95% confidence interval of the means of the above parameters of the base model before wash-in

An explicit power analysis was not done for sensitivity testing of the single and multiple coiling models as a more qualitative approach to assess suitability of the models was used.

**Replicates**

- You should report how often each experiment was performed
- You should include a definition of biological versus technical replication
- The data obtained should be provided and sufficient information should be provided to indicate the number of independent biological and/or technical replicates
- If you encountered any outliers, you should describe how these were handled
- Criteria for exclusion/inclusion of data should be clearly stated
- High-throughput sequence data should be uploaded before submission, with a private link for reviewers provided (these are available from both GEO and ArrayExpress)

Please outline where this information can be found within the submission (e.g., sections or figure legends), or explain why this information doesn’t apply to your submission:

Replicates in simulations are described in the Results or illustrated as single points in the plots

**Statistical reporting**

- Statistical analysis methods should be described and justified
- Raw data should be presented in figures whenever informative to do so (typically when N per group is less than 10)
- For each experiment, you should identify the statistical tests used, exact values of N, definitions of center, methods of multiple test correction, and dispersion and precision measures (e.g., mean, median, SD, SEM, confidence intervals; and, for the major substantive results, a measure of effect size (e.g., Pearson's r, Cohen's d)
- Report exact p-values wherever possible alongside the summary statistics and 95% confidence intervals. These should be reported for all key questions and not only when the p-value is less than 0.05.

Please outline where this information can be found within the submission (e.g., sections or figure legends), or explain why this information doesn’t apply to your submission:

Raw data is presented as points in the plots. Tests are described in the methods. F-values for ANOVA and exact P-values are described in figure legends or supplementary tables associated as Source data for each figure

(For large datasets, or papers with a very large number of statistical tests, you may upload a single table file with tests, Ns, etc., with reference to sections in the manuscript.)

**Group allocation**

- Indicate how samples were allocated into experimental groups (in the case of clinical studies, please specify allocation to treatment method); if randomization was used, please also state if restricted randomization was applied
- Indicate if masking was used during group allocation, data collection and/or data analysis

Please outline where this information can be found within the submission (e.g., sections or figure legends), or explain why this information doesn’t apply to your submission:

Not applicable as our study only involved simulations

**Additional data files (“source data”)**

- We encourage you to upload relevant additional data files, such as numerical data that are represented as a graph in a figure, or as a summary table
- Where provided, these should be in the most useful format, and they can be uploaded as “Source data” files linked to a main figure or table
- Include model definition files including the full list of parameters used
- Include code used for data analysis (e.g., R, MatLab)
- Avoid stating that data files are “available upon request”

Please indicate the figures or tables for which source data files have been provided:

The code for the models and for the figures can be accessed at https://github.com/Bui-lab/Code/tree/master/Zebrafish%20spinal%20locomotor%20circuit. Updates and revisions to the models will also be made available at this site. The simulation data that was used for the figures can be accessed at the Federated Research Data Repository at the following DOI: <https://doi.org/10.20383/102.0498>

Supplementary tables with statistical analysis has been provided as source data
